# Current state of fall prevention and management policies and procedures in Canadian spinal cord injury rehabilitation

**DOI:** 10.1186/s12913-020-05168-8

**Published:** 2020-04-15

**Authors:** Hardeep Singh, Heather M. Flett, Michelle P. Silver, B. Catharine Craven, Susan B. Jaglal, Kristin E. Musselman

**Affiliations:** 1 Toronto Rehabilitation Institute-University Health Network, 520 Sutherland Dr, Toronto, ON M4G 3V9 Canada; 2 Rehabilitation Sciences Institute, Faculty of Medicine, University of Toronto, Toronto, Canada; 3 Department of Physical Therapy, Faculty of Medicine, University of Toronto, Toronto, Canada; 4 Institute of Health Policy, Management and Evaluation, University of Toronto, Toronto, Canada; 5 Division of Physical Medicine and Rehabilitation, Faculty of Medicine, University of Toronto, Toronto, Canada

**Keywords:** Fall prevention, Document analysis, Rehabilitation, Spinal cord injuries

## Abstract

**Background:**

Preventing patient falls is a priority in tertiary spinal cord injury (SCI) rehabilitation. Falls can result in patient or staff injury, delayed rehabilitation, and hospital liability. A comprehensive overview of fall prevention/management policies and procedures in Canadian SCI rehabilitation is currently lacking. We describe and compare the fall prevention/management policies and procedures implemented in Canadian tertiary hospitals that provide SCI rehabilitation.

**Methods:**

Fall prevention/management documents implemented in SCI rehabilitation at six Canadian tertiary rehabilitation hospitals across five provinces were analyzed using a document analysis. Analysis involved multiple readings of the documents followed by a content and thematic document analysis.

**Results:**

Fall prevention/management policies and procedures in SCI rehabilitation were organized into three main categories: 1) pre-fall policies and procedures; 2) post-fall policies and procedures; and, 3) communication between and amongst staff, patients, and families. Pre-fall policies and procedures encompassed: a) the definition of a fall; b) fall risk assessments in SCI rehabilitation; and, c) fall prevention strategies. The post-fall policies and procedures included: a) recovery from a fall; b) incident reporting process; and, c) fall classification. Components of fall prevention/management policies and practices that differed between hospitals included the fall risk assessments, post-fall huddles, and fall classifications.

**Conclusions:**

Fall prevention/management is a required organizational practice for all hospitals. Although Canadian tertiary hospitals that provide SCI rehabilitation have similar components of fall prevention/management policies and procedures, the specific requirements differ at each site. There is a need for evidence-informed, consensus-driven implementation of SCI-specific fall prevention and management procedures across Canadian SCI rehabilitation settings.

## Background

Preventing falls are a patient safety priority in Canadian hospitals [1]. Hospital falls can result in injuries, delay a patient’s rehabilitation, extend length of hospital stay, and increase healthcare costs [2, 3]. Falls incidence and severity vary between clinical units [4, 5] and by patient population [6]. Rehabilitation units have higher rates of falls when compared to acute care units [4, 5]. The difference in observed fall rates in acute care and rehabilitation units may be attributed to a combination of factors, including but not limited to the patient population, environment, and clinical care goals [7]. Inpatients with spinal cord injury (SCI) in rehabilitation experience a significant rate of falls (i.e. 12.5%) [6]; yet fall prevention and management in SCI rehabilitation remains understudied.

Currently much of what is known about falls experienced by individuals with SCI is based on studies conducted in community settings [8]. Individuals with SCI encounter multiple fall risk factors including risk factors that pertain to SCI-related impairments and/or the activities an individual engages in [8, 9]. Since traumatic SCI tends to be more prevalent in middle-age [10], individuals with SCI tend to be younger in age than other neurological populations and have unique rehabilitation needs [11]. Fall prevention/management initiatives should be tailored to address their unique fall risk [9]. In order to effectively prepare individuals with SCI for falls, effective, and targeted fall prevention should begin while an individual is in SCI rehabilitation [12, 13].

Previously, we completed semi-structured interviews with administrators regarding the challenges they experienced when implementing fall prevention/management policies and procedures in SCI rehabilitation. Administrators perceived that the acute care fall prevention/management policies and procedures applied in Canadian tertiary hospitals that provide SCI rehabilitation failed to account for the specialized fall prevention/management needs of patients with SCI [12]. Fall prevention/management tools are often applied to patients with SCI with limited context-specific or population-specific validation [12, 14]. In this study, we aimed to generate a comprehensive understanding of the fall prevention/management policies and procedures that are applied to patients with SCI in Canadian rehabilitation hospitals. Prior to validating or critiquing current policies and procedures, the authors aimed to describe and compare the fall prevention/management policies and procedures in place at Canadian tertiary hospitals that provide SCI rehabilitation.

## Methods

This is a qualitative descriptive document analysis [15]. Ethical approval for this study was obtained from the Research Ethics Board of the University Health Network. Informed written and verbal consent were obtained from all study participants.

### Data collection

We contacted at least one administrator from all 15 Canadian rehabilitation hospitals that provide specialized rehabilitation services to patients with SCI using snowball sampling [12]. The administrators that consented to participate in our research study were asked to provide any documents from their affiliated tertiary rehabilitation hospital that related to the assessment, prevention, tracking, risk management, and/or education of falls and were directly or indirectly relevant for patients with SCI. The documents were received in an electronic or hardcopy format. We collected documents from six Canadian tertiary rehabilitation hospitals that operate within a universal payer health care system. The length of stay in these specialized Canadian rehabilitation facilities ranged from 50 to 124 days [16].

### Data analysis

We conducted a document analysis of the fall prevention/management documents used in SCI rehabilitation. A document analysis was an appropriate method pertinent to the study aims- to describe and compare the fall prevention/management policies and procedures implemented for patients with SCI within different tertiary rehabilitation hospitals. The analysis was guided by a document analysis approach [15] wherein a document analysis is a combination of a content and thematic analysis. The analysis involved a superficial examination of the content, followed by thorough readings of the text. The superficial examination allowed the reviewer to gain familiarity with the content (e.g. titles, headings/subheadings, sections, references) and presentation (e.g. format, length) of each document. Next, the text was read thoroughly, and inductive descriptive codes were generated based on the document content. The data were then organized into these aforementioned descriptive codes. Lastly, similarities and differences between the policies and procedures of tertiary rehabilitation hospitals were identified [15]. The policies and procedures were considered similar if they used the same definitions, required the same actions/steps to be executed, used the same tools, and/or had comparable time requirements for procedures. Any similarities or differences noted were described in the results.

## Results

Twenty-eight fall prevention/management documents were reviewed from the six participating Canadian tertiary rehabilitation hospitals from five Canadian provinces. Documents included fall prevention interventions/strategies for staff and patients (8), fall risk assessments (7), fall prevention and incident reporting policies (6), post-fall algorithms (3), post-fall procedures (2), a communication tool (i.e. patient handling and moving sign) (1), and a fall prevention pamphlet (1) (see Table 1). Although we aimed to collect information on fall prevention/management policies and procedures that applied to patients with SCI in a rehabilitation hospital, we found that SCI-specific policies or procedures did not exist at any of the six sites. Fall prevention policies and procedures were created at an organizational level and applied in all hospital units (e.g. acute and rehabilitation).

**Table 1:** Description of facility structure and number of fall prevention documents provided

Code	Facility structure	Number of documents provided
Site A	Free-standing academic SCI rehabilitation facility	3
Site B	SCI program imbedded in Health Sciences Centre	6
Site C	Free-standing academic neurologic rehabilitation facility	5
Site D	Free-standing academic rehabilitation hospital with regional SCI program	9
Site E	Spinal cord injury program imbedded in City Hospital	3
Site F	Free-standing academic rehabilitation Hospital with SCI program	2

**Fig. 1:**
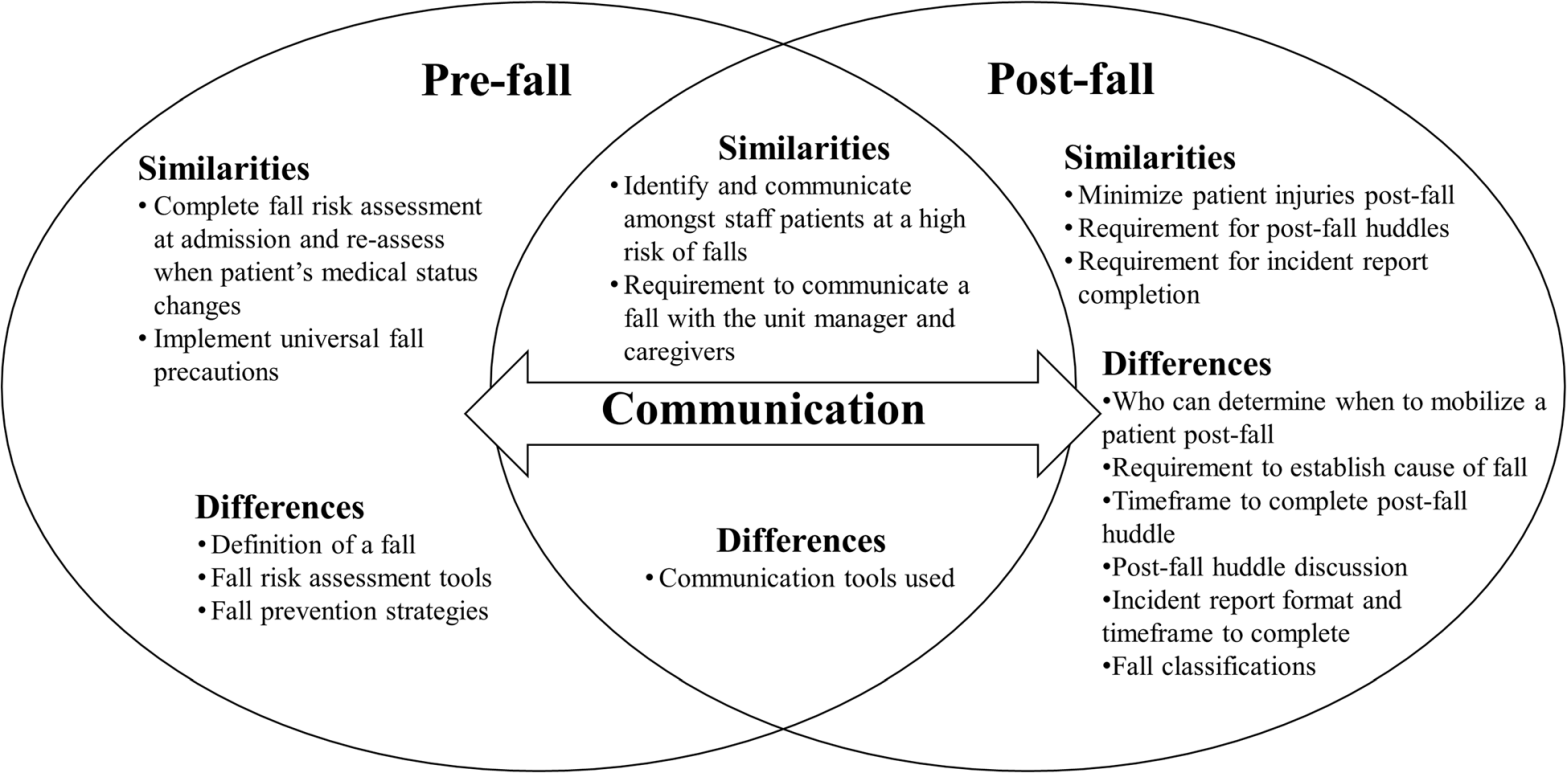
Similarities and differences in the pre-fall, communication, and post-fall policies and procedures in Canadian tertiary hospitals that provide SCI rehabilitation

Findings from the document analysis were organized into three main categories. These included: (1) the pre-fall policies and procedures, (2) the post-fall policies and procedures, and (3) communication (see Fig. 1). The first two categories organized the information into sequential components that would occur prior to and after a patient fall. Communication amongst and between staff, patients, and caregivers was an essential component of both the pre-fall and post-fall policies and procedures (see Fig. 1). The subthemes were formed from the inductive codes identified from the document data.

### Pre-fall policies and procedures

This category encompassed the fall prevention policies and procedures from each site that were implemented prior to the occurrence of a patient fall.

#### Definition of a fall

Not all sites included an unambiguous definition of what constituted as a fall in their documents. Sites A, B and F did not provide a definition of a fall within their fall prevention documents. Sites C, D and E defined a fall as, an unintentional or unexpected event that resulted in a person “coming to rest on the ground, floor or other lower level, with or without an injury.”

#### Fall risk assessments in SCI rehabilitation

The document analysis revealed that all units were required to assess fall risk upon a patient’s admission to an inpatient unit. The following instruments were used to assess the risk of falls for patients with SCI on the rehabilitation units: the STRATIFY [17], Schmid Fall Risk Assessment Tool [18], the Morse Fall Scale [19], and three customized fall assessment tools developed by the institutions (see Table 2). At all sites, the fall prevention policies mandated that clinical staff were to reassess the patient’s risk of falling after a fall occurred, or when there was a change in medical status. No site defined what constituted as a change in status, rather it was left open to interpretation by the clinical staff.

**Table 2:** Description of fall risk assessment tools used in SCI rehabilitation settings. Column three represents the maximum score (a high score infers a higher fall risk). The thresholds for assigning fall risk based on the scores are specified in column four

Scale Name	Domains Evaluated	Maximum Score	Interpretation
STRATIFY [17]	recent falls, agitation, vision, toileting frequency, transfers and mobility	6	0 = low fall risk 1 = medium fall risk 2 + = high fall risk
Schmid Fall Risk Assessment Tool [18]	mobility, mentation, elimination, prior fall history and current medications, agitation, attempting to get out of bed unsafely, vision, orthostatic hypotension, balance and sensory issues, history of fractures or osteoporosis, alcohol/substance abuse and malnutrition	5	0–2 = normal fall risk ≥3 = high fall risk
Morse Fall Scale [19]	fall history, secondary diagnosis, ambulatory aid, IV, gait/transfers, and cognition	125	0–24 = low fall risk 25–44 = moderate fall risk ≥45 = high fall risk
Customized Scale Site D	history of falls, medication, dizziness, sensory impairments, toileting, cognitive impairments, balance/mobility issues, co-morbidities, bed transfers/mobility, mobility in patient room, bathroom and on the unit, and behavioural traits (e.g. judgement, self-control/impulsivity, anxiety)	Yes or No scale	Any yes answer requires development of a plan
Customized Scale Site E	neuromuscular deficits, cognition, sensory deficits, bowel/bladder, postural hypotension, history of seizures	17	0 = low fall risk ≥1 = high fall risk
Customized Scale Site F *based on the Morse fall scale	number of diagnoses, vision, toileting, medication, mobility, and cognition	100	0–64 = low fall risk ≥65 = high fall risk

#### Fall prevention strategies

Table 3 outlines fall prevention strategies that were referenced in each site’s fall prevention documents. Fall prevention strategies were categorized into strategies pertaining to the environment, communication/education, and interprofessional assessments. Universal fall prevention strategies were initiated prior to a fall, but remained in place for the duration of a patient’s hospital stay regardless of changes in function, mobility status, and medical condition.

**Table 3:**
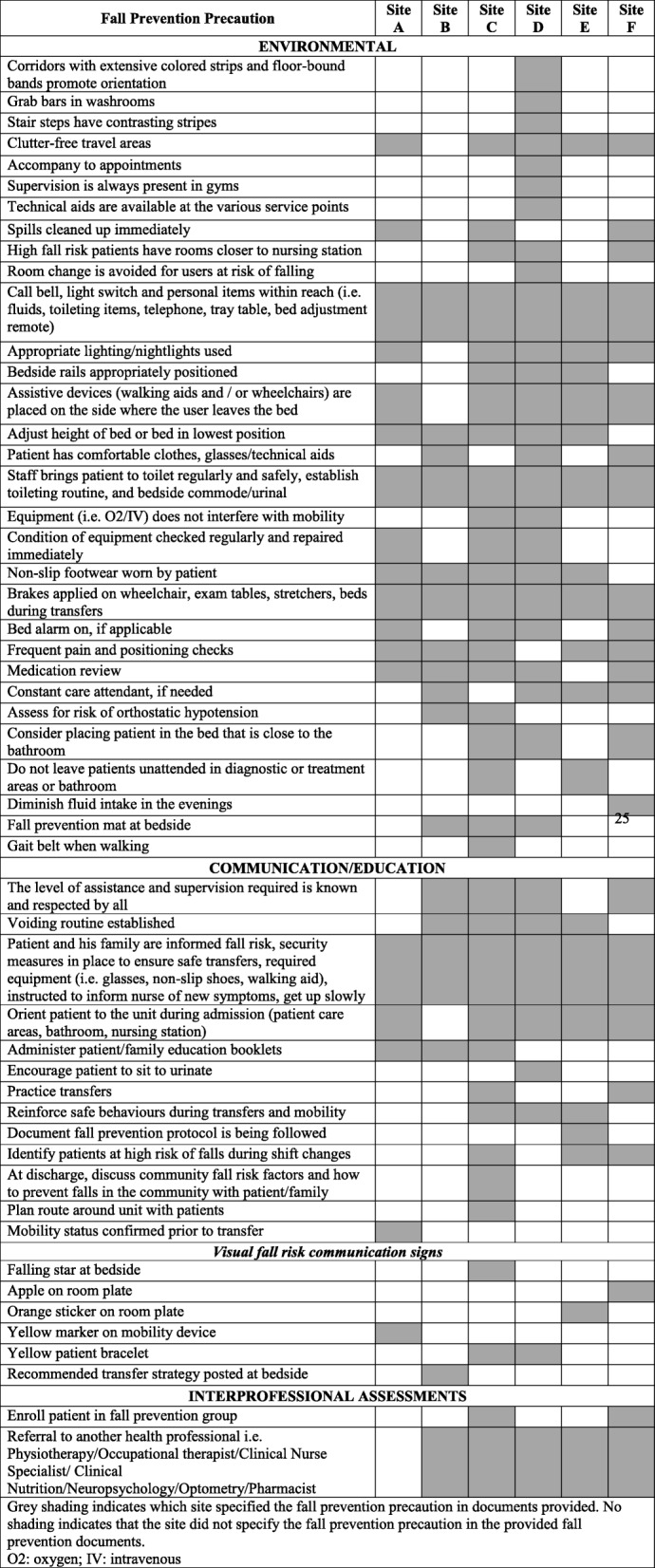
Fall prevention precautions cited in fall prevention/management documents at each site

Grey shading indicates which site specified the fall prevention precaution in the documents provided. No shading indicates that the site did not specify the fall prevention precaution in the provided fall prevention documents

*O2* oxygen, *IV* intravenous

### Post-fall policies and procedures

Post-fall policies and procedures included elements of fall prevention and management that were implemented after a patient experienced a fall.

#### Recovery from a fall

After a patient falls, clinicians at all sites were required to perform a thorough clinical assessment of the patient (e.g. neurological assessment, vitals, level of consciousness and cognition, and injuries sustained), and the environment (e.g. location of fall, environmental hazards). All policies mandated that the assessment was to be completed while the patient was still on the floor, to avoid exacerbating a potentially serious injury. Site D’s policy specified that a nurse practitioner or physician would determine if a patient could be mobilized safely. Other sites’ fall management policies did not specify who would determine if the patient was safe to be mobilized. To prevent staff injuries, sites specified that if a patient could not perform an independent transfer, a lift transfer was required. Site B’s fall management policy clarified that the reason for using a transfer lift was to protect the safety of the staff. Site A instructed that if a severe injury was sustained after a fall, the patient must be transported to the emergency department for treatment. Sites A, C, D, and E required the cause of the fall to be established. Site A further specified that the cause of the fall should be documented in the patient’s record. Interestingly, only site A’s fall management policy stipulated that after a fall, the staff were to consult the patient on their perception of the causal factors when appropriate.

#### Post-fall huddles

Site A required the clinical director to hold a meeting where details of the fall were reviewed with key staff members. For critical or severe falls, Site A required a unit-level debrief to take place within 14 days of a patient fall. Site B required the post-fall huddle to take place within 15 min of the patient fall. Site B’s post-fall huddle included all staff involved in the patient’s care, the patient, and their family members. Site B’s policy had a formal post-fall huddle, in which the following questions were proposed to facilitate discussion: 1) Why did the patient fall? 2) Was the patient at a correct fall risk level? 3) Was the patient identified as having a high risk of falling? 4) Were the appropriate interventions put in place? 5) What are we going to do differently in our care for this patient? The results of the post-fall huddle were documented in the patient’s medical chart. At site C, falls were to be reviewed at the next team rounds meeting. At site E, a post-fall safety huddle was to be completed by the next working day to discuss the fall details and create an intervention. No formal post-fall huddle procedures were reported in the documents provided by sites D and F.

#### Incident reports

Sites A and C specified that incident reports were to be completed within 24 h of a patient falling. Site C’s incident report form asked the person who completed the form: “What more could be done to prevent similar falls/injury from occurring? Analysis: Why do you think they fell in spite of all the prevention efforts in place?” Site E utilized a paper-based incident report, but required staff to report falls incidents via telephone to the “safety line”. Documents obtained from sites B, D and F did not include details of their incident reports.

Incident reports were shared among several members of the healthcare team at each of the sites. Incident reports at site A were shared with the clinical director, manager, and the Falls and Quality Control Committees. At site D, the incident reports were shared with the unit manager, physician, pharmacist, and the program director. Documents revealed that only site A and D organized quality improvement committees that conducted reviews of the incident reports to identify areas for improvement.

#### Classification for a fall

All sites used different approaches to classify the type of fall. Site A’s incident report required reporting of a near miss. That is: “Incident occurred but did not reach any person(s)”. At Site A, falls were classified as “critical,” “severe,” “moderate,” “minor,” “near miss (potentially severe),” or “near miss”. Site B used the following classification: “no injury,” “apparent/suspected injury”, and “apparent/major injury”. Site C classified falls as: “near miss,” “no apparent injury,” “slight no treatment,” “slight minor treatment,” “moderate”, and “serious”. Documents provided by sites D, E and F did not include their classification approach.

### Communication

Various forms of communication were a required element in the pre-fall and post-fall policies and procedures (see Table 3). Communication prior to a fall involved sharing a patient’s level of fall risk with staff members involved in the patient’s care at all sites. The use of communication tools varied between sites. Communication tools used to identify an increased fall risk included: an orange sticker on the room plate (site E), a yellow marker on a mobility device (site A), an apple (site F), or yellow bracelet (sites C and D), and verbal updates during nursing shift changes (site C). In addition to visual signage, site E also recommended consulting with the Fall Reduction and Injury Prevention Coordinator (FRIPC) for complex patients that presented with multiple fall risk factors. The FRIPC assisted in establishing the root cause of the fall and delivered individualized patient fall prevention education.

All rehabilitation sites referenced the provision of patient education materials. Sites A and E specified that the patient/Substitute Decision Maker (SDM)/caregiver should be provided with written education on the risk of falls prior to a fall. Fall prevention education was to be reinforced with the patient/SDM/caregiver after a fall. Others did not specify a format to deliver the fall prevention education to the patient and/or caregivers.

The documents revealed that the unit manager and the patient’s substitute decision maker/family were to be notified immediately after a patient had fallen at all sites. Further, if a patient experienced a fall, that information was to be communicated amongst all staff members on the unit.

## Discussion

This is the first study to describe and compare the fall prevention/management policies and procedures implemented in Canadian SCI rehabilitation settings. A comprehensive understanding of fall prevention/management policies and procedures can supplement our understanding of the implementation challenges recently raised by administrators in SCI rehabilitation [12]. Components of fall prevention/management were organized into three categories: pre-fall policies and procedures, post-fall policies and procedures, and communication. Similarities and differences between sites pertaining to each of these categories were described (Fig. 1). Findings from this study extend prior fall prevention literature [20, 21], which has primarily focused on fall prevention/management in acute care settings. Our findings revealed that all sites had organization-wide, rather than SCI-specific, fall prevention/management policies and procedures. In addition, all sites required a fall risk assessment tool to be completed with each patient at admission and implementation of the related fall prevention strategies. Differences were found in pre-fall policies and procedures (e.g. which fall risk assessment tools were used, and the recommended universal precautions), post-fall policies and procedures (e.g. requirements for post-fall huddles, classification for falls, and requirements for incident reports) and fall prevention/management communication (e.g. specific communication tools used). These differences are likely due to the lack of research evidence and best practice guidelines in these areas. In order to generate more effective and appropriate fall prevention/management policies and procedures more research is needed to determine how population-specific fall prevention needs of patients with SCI can be considered in SCI rehabilitation.

Our findings confirm that the policies and procedures implemented in SCI rehabilitation are consistent with the Required Organizational Practices (ROPs) for fall prevention that are assessed by Accreditation Canada [1]. Accreditation Canada is an organization that evaluates a healthcare organization’s adherence to ROPs, which are evidence-based organizational practices that aim to enhance patient safety and minimize risk [1]. These practices include a requirement to assess fall risk, and report and track fall incidents, as well as a post-fall procedure for reviewing the details of each fall.

Our results indicated that fall prevention/management policies and procedures in SCI rehabilitation were similar to those implemented in acute care settings [22]. Fall prevention/management policies and procedures from the organizations sampled were formed at an organizational-level, as there was limited evidence for unit-level or population-specific fall prevention/management strategies [3, 23]. However, it has been suggested that the current fall prevention evidence fails to support the specialty needs of rehabilitation, and specifically SCI rehabilitation [12, 23]. Higher rates of falls in rehabilitation settings compared to acute care are suggestive of a need for high quality fall prevention research in rehabilitation units.

Not all fall prevention policies reviewed in this study included an explicit definition of a fall. Without a consistent definition of a fall, interpretations of what constitutes a fall can differ between patients and clinicians [24] and could lead to underreporting falls as well as missed opportunities for delivery of fall prevention/management education to patients. This suggests a need for a clear definition of a fall in SCI rehabilitation. An explicit and consistent definition of a fall at all hospitals will facilitate consistent reporting practices and accurate comparisons between hospitals and rehabilitation settings [25].

While not all sites explicitly defined a fall, the sites that did define a fall had used a definition that was consistent with the widely accepted definition of a fall from the Canadian Patient Safety Institute. The Canadian Patient Safety Institute defines a fall as: “an event that results in a person coming to rest inadvertently on the ground or floor or other lower level, with or without injury” [26]. Further, a near fall is defined as: “a slip, trip, stumble or loss of balance such that the individual starts to fall but is either able to recover (witnessed or unwitnessed) and remains upright because their balance recovery mechanisms were activated and/or caught by staff/other persons, or they were eased to the ground or floor or other lower level, by staff/other persons” [26]. Only one site included an explicit definition of a near fall; however, it was vaguely defined. It is important to note that the definitions of a fall and near fall fail to distinguish between a controlled fall during therapy and an uncontrolled fall, and the definition of a near fall is similar to the actions that occur during a controlled fall. A lack of clarity of what constitutes an unexpected fall/near fall, versus a fall/near fall during therapy in a supervised setting was raised as a challenge by administrators [12]. Administrators believed experiencing controlled falls in a supervised setting was a training technique/therapeutic intervention for patients with SCI to learn their new tolerances, functional and physical abilities, and practical fall prevention skills [12]. A controlled fall that is part of therapy is different than an unintended or unexpected fall during therapy and this distinction is not recognized in the policies/procedures of the sites or in the widely accepted definition outlined above [26]. In order to support a practical approach to fall prevention training in SCI rehabilitation [9, 27], the widely accepted definition of a fall/near fall must differentiate between a controlled therapy fall versus an unexpected/unintended fall.

Another difference we noted between fall prevention/management policies and procedures in SCI rehabilitation settings was related to the classifications of falls. A classification of a fall is needed to understand the root causes, and to tailor interventions [23, 28]. Inconsistent fall classifications can be a barrier to comparing falls between organizations. Not all rehabilitation hospitals differentiated between whether a fall was preventable (i.e. if it could be anticipated by staff) or not. This highlights the need for future research to establish an agreed upon classification system of fall etiology and severity in tertiary rehabilitation settings.

The lack of direction for assessing the risk of falls in rehabilitation settings [23] is demonstrated in our analysis. For instance, all sites used different risk assessments. The purpose of a risk assessment tool is to assist clinicians in identifying sub-groups of individual patients at high risk of falling [14]. Fall risk assessment is a required practice for healthcare organizations to achieve accreditation [1]. However, there is no clear evidence-based direction for rehabilitation units to determine which tools are best suited to reduce falls in SCI rehabilitation and when to re-assess a patient’s fall risk. During a patient’s stay in SCI rehabilitation, their risk of falling may change as their physical function changes (i.e. progression from wheelchair to walking). To account for the change in fall risk status, it is necessary to re-assess their risk of falling on an ongoing basis. Applying risk assessment tools in a context inconsistent with the context in which they were developed, can lead to inaccurate results [29]. Our findings indicated that the fall risk assessment tools (e.g. STRATIFY, the Schmid, and the Morse Fall Scale) that were used to assess fall risks in patients with SCI in rehabilitation were the same tools used in acute care units with different patient populations [30]. However, these assessments were previously found to poorly predict fall risk in rehabilitation settings [29, 31, 32]. As well, some SCI rehabilitation hospitals in the current study used customized fall risk assessments, but some of these tools lack psychometric standardization [14]. Further, it has been suggested that the use of risk assessment scores to determine fall risk in rehabilitation is an ineffective use of staff time due to the lack of useful information produced [31]. This may explain the poor staff adherence in completing the risk assessment tools reported by administrators working in SCI rehabilitation [12]. Instead, in rehabilitation settings, some studies suggest it may be more appropriate to assume all patients are at a risk for falling or base the fall risk assessment on clinical judgement [31]. For example, a study from a geriatric rehabilitation unit found clinical judgment had higher accuracy in predicting falls, than risk assessment tools [31]. Future research should continue to explore how to best assess the risk of falling for patients with SCI in rehabilitation settings where they are encouraged to mobilize and progressively increase their independence.

The fall prevention/management documents suggested all falls, including near misses, were to be reported in all Canadian tertiary hospitals that provide SCI rehabilitation. In our previous study, SCI rehabilitation administrators perceived that near misses and no-harm falls that occurred during therapy, were under-reported [14]. Issues with reporting falls include nurses’ beliefs that there is no value in reporting near misses and a lack of time to complete complex incident reports [33]. These issues may be addressed by educating staff on the importance of tracking falls, reinforcing a “no-blame reporting of incidents”, and reducing lengthy reporting processes [34]. Also, the use of wearable devices to detect falls in this population group could be considered as a future direction [35–37].

Our document review suggested that falls in SCI rehabilitation were regularly tracked on incident reports and discussed in post-fall debriefs. In addition to informing individualized fall prevention plans [23], incident reports can inform fall prevention improvement efforts within the parent organization. For example, the analysis of incident reports could identify fall hazards and identify the targets for fall prevention initiatives [25].

Communication is a pivotal aspect of fall prevention. Communication strategies varied between tertiary rehabilitation hospitals, which suggests a lack of agreement on the most effective communication methods. Visual signage is often used to identify a patient with a high risk of falling. Previous literature has recognized that communication gaps were shortcomings in effective fall prevention [38]. Depending upon the type of signage and how it is used, it is important to note certain signs may “blend into the background” [39]. To address this challenge, a fall prevention toolkit with standardized communication resources was created [20]. The toolkit has been pilot tested in four medical facilities in the United States and found to reduce falls and improve communication among staff and patients [20].

The findings of this study are limited by the documents provided by each facility. Sites were instructed to provide any fall prevention/management documents relevant to patients in their SCI rehabilitation programs. A potential bias in reporting could be due to failure of sites to provide all of the relevant documentation. A sampling bias may exist as we used snowball sampling to recruit administrators who provided access to fall prevention documents from their affiliated hospital. Volunteer bias should also be considered as it was not feasible to include all Canadian tertiary hospitals that provide SCI rehabilitation in this study. Nevertheless, valuable insights into the fall prevention practices within six Canadian tertiary hospitals that provide SCI rehabilitation across five provinces are described herein. Future research should explore how to best predict fall risk in this population, effectively analyze fall data and learn from fall trends, deliver fall prevention education as well as identify the prevalence, predictors and outcomes of falls among patients with SCI in tertiary rehabilitation hospitals. More research examining the effectiveness of fall prevention interventions and strategies among learning organizations in SCI rehabilitation is needed.

## Conclusions

This study described fall prevention/management policies and procedures implemented in six Canadian tertiary hospitals that provide SCI rehabilitation. Fall prevention is a high priority in tertiary rehabilitation hospitals. These hospitals share common aspects in their pre-fall and post-fall policies and procedures, as well as fall prevention communication. However, inconsistencies are noted in aspects of fall prevention, that were lacking supporting research evidence. This included differences between sites in the type of fall risk assessment tools used, fall prevention precautions implemented, communication tools used, requirements for post-fall huddles, format of incident reports, and classification of falls. Findings from this study highlight a gap that there are no SCI-specific fall prevention/management policies and procedures in Canadian tertiary hospitals that provide SCI rehabilitation. There is an urgent need for a common nomenclature across sites for describing fall type, location of the fall (i.e. washroom versus therapy setting), severity of the fall (i.e. loss of consciousness), and associated injury/ies (i.e. no injury, mild, moderate or severe injury) to inform fall prevention/management and auditing strategies in SCI rehabilitation.

## Data Availability

The datasets generated and/or analysed during the current study are not publicly available due to confidentiality of sites, but are available from the corresponding author on reasonable request.

## Abbreviations

SCI: Spinal cord injury
FRIPC: Fall Reduction and Injury Prevention Coordinator
ROPs: Required Organizational Practices
SDM: Substitute Decision Maker
